# Illinois State Dental Society

**Published:** 1887-06

**Authors:** E. E. Cady


### Reports of Society Meetings.

### ILLINOIS STATE DENTAL SOCIETY.

Reported for the Independent Practitioner by E. E. Cady, D. D. S.

The twenty-second annual meeting of the Illinois State Dental Society was held in the hall of the Y. M. C. Association, in Jacksonville, May 10-13. The society numbers 129 members, a large majority of whom were present, with many dentists from abroad. The meeting was a most pleasant and profitable one, both from a professional and social stand-point. The papers were interesting and the discussions enthusiastic. Dr. G. V. Black was present with his apparatus for the culture of microbes, and with a large number of tubes and slides containing, in different stages of development, microscopical fungi of many varieties. His lectures were one of the most important features of the meeting. Invitations from the Superintendents of the different State institutions located in Jacksonville were accepted, and at different times the Deaf and Dumb, Blind and Insane Asylums were visited by the society in a body.

TUESDAY, MAY 10th—MORNING SESSION.

After reports of the various officers and committees, the President, Dr. W. T. Magill, of Rock Island, delivered the annual address. The past year, he said, had been one of great advancement in dentistry, particularly in the west. Two new dental journals had made their salutatories from that section, and in our own State four new local or district societies had been formed. Mention was made of the Younger operation of implantation, of the Knapp blow-pipe, and of Dr. Herbst's visit to this country, and the expositions of his method of operating. Attention was called to the deplorable ignorance of dental subjects, and particularly dental hygiene, among people well educated in nearly everything else, and the necessity for proper teaching in the public schools urged as a remedy. Occasional lectures by incompetent teachers were not sufficient, but approved text books should be used, and the subject, as far as necessary for this purpose, taught thoroughly.

### AFTERNOON SESSION.

A vote of thanks was given to the committee who had so thoroughly performed the work of arranging district societies in accordance with the resolution of last year. It was reported that, in addition to the societies already organized in this State (the Illinois State Society, Central Illinois Society, Chicago Dental Society, Chicago Odontological Society and Chicago Dental Club), four new ones had been formed, viz.: Southern, Northern, Western and Eastern District Societies, with an aggregate membership of 125.

Dr. Black then spoke as follows:

It is difficult for most men to fathom the depths of the subject of micro-organisms and their work. This is owing somewhat to early training, and to the fact that the general trend of thought has long been in a different direction. Experimentation in this line is confined to a few persons, and upon them we are compelled to rely for our information. Notwithstanding the many disadvantages, this subject is progressing rapidly, and our knowledge each day is increasing.

A misconception exists in the minds of those who say a thing "spoils." A piece of meat laid in the sun is said to spoil. This is not true. Beef broth exposed to a favorable temperature will, it is said, spoil. I say it will not. I might thus go on with a whole category of things which are said to spoil, but which in reality do not. *They are spoiled.* If let alone they will last till the end of time. Chemical changes are never inaugurated within the beef or the broth except through external or outside interference. There are various forces active in matter, but the great disturbing force is life; what life is we have not determined. It is different from all other forces, in that it brings the elements together for its purpose.

A dog, capable of going about, gathers that material which is best suited for its own particular use, and just so do all other forms of animal and plant life choose sustenance. The dog dies, but the carcass remains forever if let alone—if no other life interferes with it. This is the essential point: You say this broth spoils. No! It *is* spoiled; wherever we find a chemical change, there a living being has had its growth.

Food taken into the stomach is converted into other chemical forms by digestion. Thus we have assimilation or tissue building, de-nutrition or formation of waste products, and reproduction. These processes, these changes are found in every form of life, from the greatest to the smallest, in vegetables as well as animals. They occur in the plants in this broth, and in the case of the dog, this is the process by which his body is destroyed.

The apparatus for the culture of microbes was then shown, and five inoculations in sterilized broth were made from the mouths of members, two being from beneath artificial plates, one of which was rubber and the other gold.

The paper of Dr. N. J. Roberts, of Waukegan, Ill., was then read, his subject being "Regulating Appliances," and several models were shown illustrating his method. The speaker prefaced his remarks by saying that the forceps, judiciously applied, and at the proper time, is the best regulator we have in four cases out of five, and that the removal of the first permanent molars just prior to the eruption of the second bicuspids, will prevent irregularities in one-half of the cases where there seems to be a predisposition to malformation. Consequently, the forceps is the first regulating appliance to be used, and other methods when that fails.

Nature does her best to bring the teeth into line, and fails only from lack of space, or other obstructions. The teeth are developed independently of the maxillary bone, and the principal cause of their deformities is the intermingling of races. Children often inherit the large bones of one parent, with the small teeth of the other, and *vice versa*. This being understood, it will readily be seen that much regulating can be done by extraction.

Dentists usually consider regulating cases undesirable, and when they are compelled to undertake them seek the simplest appliance possible. So much time is required in making and looking after an apparatus of the usual order, that one with an extensive practice can hardly afford to undertake these cases. Some of the leaders in this branch undoubtedly construct a set of screws and bands which do the work satisfactorily, but to the inexperienced their manufacture is so formidable that the great body of the profession will never adopt them. The popular demand is for something nearly automatic in its action, and easy of construction. The Coffin and Talbot springs are both good in their place, but cases where they can be employed advantageously are limited. In fact, this is true of all appliances now in general use, and where an extensive operation is to be made, a constant succession of plates, appliances and principles, is required to meet the new condition of things.

Experiment shows that a force of fifteen pounds can be obtained from the spring of black rubber, while the largest piano-wire bends at a strain of three and one-half pounds. This being the case, black rubber seems a desirable material, and I shall describe a regulating appliance made from it. A plate is made of thin black rubber with a thick shoulder opposite the teeth to be forced outward. Where it is desired to keep the jaws apart, a cap of the plate material should extend over one or two teeth on each side, and up the buccal surface, so that screws can be inserted to hold it firmly. Opposite the teeth to be forced out the plate should be cut with a saw, the length of cut and width of section to be determined by the amount of force desired. Screws should be set in the plates opposite the teeth to be drawn in or rotated, and to these ligatures attached.

If a screw is inserted in the section opposite the tooth, when it is turned it will tend to force the section onward, and the tooth will be driven out. A ligature attached to the tooth, and a screw placed in the palatine side of the plate, will draw the former in. Rotation can be accomplished in the same way, by banding the tooth to be turned and having on the band, at the proper place, an arm for attachment of a rubber ligature. Where enough spring force cannot be obtained from the plate, the Talbot wire spring may be used to good advantage. When the tooth is forced into the required position, a screw may be so placed as to hold it there.

The backward or forward motion may be obtained by making a short arm on the plate, and using a spring or elastic band. An appliance of this nature is always held firmly by turning up the screws in the caps before referred to. The How crown screws, with their ends squared to fit a watch key, are admirable for this purpose. Use a jeweler's adjustable key, and give one to the patient.

It is well to place a drop of cement on teeth carying ligatures, to prevent the latter from impinging on the gum. This may be done also in the case of superior cuspids to be forced out, the shape of the tooth sometimes acting as an inclined plane, not holding the screw securely.

The advantages claimed for this system are simplicity, cheapness and near approach to automatism. During the past two years the essayist had employed it in over thirty cases, with great satisfaction.

DISCUSSION.

*Dr. E. S. Talbot*—I do not believe in the statement made by the essayist, that four cases out of five of the irregularities presented to us can be remedied by the use of forceps. On the other hand, I am convinced that more irregularities are produced by injudicious extraction than by any other cause. Teeth should not be extracted early.

We must consider the maxillæ as composed of two distinct bodies —the jaw-bone proper and the alveolar process—and consequently the entire structure has two functions. The true bone is simply for the attachment of muscles, while the alveolus is solely for the purpose of retaining the crypts of the teeth while forming, and their retention when erupted. In adult life, the alveolar process changes three distinct times, and while the jaw-bone proper grows entirely independent of the teeth and alveolar process, the latter for its development depends entirely upon the two former. This the speaker illustrated upon the blackboard.

If first teeth are extracted the process contracts, and if the second do not make their appearance for some time they have not sufficient room to come into line with their neighbors, and are crowded to one side or the other. The temporary teeth being removed, the first permanent molar works forward into the space of the second temporary molar. I have found in such cases that the permanent tooth has moved one-eighth to three-fourths of an inch. Extracting, as a rule, is simply opening the door to irregularities, and I do not think it pardonable except in extreme cases. Extraction of the temporary molar often causes the holding up of the permanent cuspid, and prevents its falling into place. I believe the screw principle, as adopted by Dr. Roberts in the models here shown, a good one.

*Dr. Black*—Premature extraction of temporary teeth, in many cases, gives rise to irregularities. If the anterior teeth are removed, those back move forward, and this occurs to such an extent that I have seen the wisdom teeth moved forward to the place of the bicuspid. It is erroneous to be governed in this matter by size of the teeth or arch, as the latter can be enlarged. We should be guided almost entirely by the features. If the face is flat, the arch should be enlarged and the teeth brought out. If the face is prominent and the features round, extraction may be admissible.

*Dr. Kitchen*—Related the case of a boy where irregularities were corrected by filling the molar teeth in such a manner as to keep the jaws apart.

*Dr. Black*—There is no necessity for keeping the jaws apart. I have recently moved out three superior incisors, where the occlusion was undesirable, without separating the jaws. My usual method is to fasten wires to the molar teeth by bands. To these wires I attached ligatures of rubber dam. I do not use the tubing, as it is much more clumsy than the dam, and will not wear so long, besides having less strength.

*Dr. Newkirk*—I use the rubber dam in preference to the tubing, and find it as Dr. Black has stated. I agree with Dr. Talbot in condemning the use of forceps, and think that more false or misleading precepts could not appear upon the pages of our report. It is true that one is often tempted to extract, for it frequently appears as if the removal of a single tooth would remedy the entire trouble. This is a mistaken idea. In so doing we rob Peter to pay Paul, and the former will in due time come in for his pay, with interest. Extraction, in these cases, is usually a mere temporary make-shift, which will work serious injury in after life. The future changes must always be taken into account before extracting either temporary or permanent teeth. It is a mistake to suppose that if we extract a temporary tooth the alveolar process will remain for its successor. That tissue is formed and exists for the express purpose of covering the teeth, and when the latter go the process soon follows.

Regarding the models here shown, while it is true that simple appliances are the best, Dr. Roberts has not apparently carried out his own idea. He certainly attempts to do too much with one plate. Cases of the nature here shown require several. In this mouth the cuspids should be moved first, then a new plate made and the laterals moved. I should not want to have such a complicated appliance in my own mouth; moreover, it is too thick; there is too much of it, and the whole piece too rough and improperly finished. The bands used are too thick and heavy. Teeth close together would require separating before it could be adjusted.

*Dr. Stevens*—In the absence of Dr. Roberts, my interpretation of his essay is that he moves the permanent teeth (not the temporary) with forceps at one operation, instead of taking six or eight months for it. As for this apparatus being clumsy, I think Dr. Roberts merely intended to show us in one plate all the different methods he uses, and do not believe he makes a practical case with so much wire, etc.

*Dr. Noyes*—Said that in addition to the anterior movements of the posterior teeth referred to, the anterior teeth moved backward a little, and related a case in point. The value of Dr. Black's suggestion regarding the features could hardly be overestimated.

*Dr. Stevens*—Dr. Newkirk, did I correctly understand you to say the jaws contract?

*Dr. Newkirk*—No, sir. I said the alveolus.

*Dr. Gardner*—I believe extracting far better than enlarging the arch in some cases. I do not believe the arch contracts or recedes. Regulating may be done at any age—fifty or sixty years. In making regulating appliances I use silver or copper and soft solder, as it costs less and they are more easily made.

*Dr. Sitherwood*—I have had two cases of necrosed bone from undue retention of temporary teeth. While the general principle of non-extraction is correct, there are cases in which it is necessary. I do not have good success with plates, nor have I been successful with the appliances of Dr. Farrar. In one case much trouble was caused by the patient screwing the appliance up too tight. I never take a case of regulating unless I am confident that the patient will visit me when directed, and wear any appliance that I may think necessary to put in the mouth. The matter of ability and willingness to pay must also be taken into account.

*Dr. Reid*—In making these appliances, I have employed ordinary nickel, instead of gold or platinum. The metal may be rolled down as thin as needed; the bands are easily made and may be soldered with soft solder.

*Dr. McKellops*—What is the objection to gold?

*Dr. Reid*—The nickel is more easily manufactured and soldered.

*Dr. McKellops*—It is easy to say what can be done, but the proper way to demonstrate the truth of such a matter is to bring in the material and do the work before the society. Should this be done, I feel confident of proving my ability to manipulate gold for these purposes as easily as anyone can use any other metal. I do not believe Dr. Roberts intended to convey the idea that he moved teeth with forceps. I believe in extracting when necessary, and also in the use of good practical judgment and common sense in the different cases that present.

*Dr. Stevens*—Then you do not believe in Dr. Younger's theory?

*Dr. McKellops*—I believe the use of forceps in regulating would break up the nerve connections, and should not be practiced. We do not do Dr. Coffin justice in this country. I saw fifteen hundred of his practical cases while in Europe, and his method is certainly admirable. I cannot understand why dentists will not use coin gold for all appliances in the mouth, in preference to any base metal. It cannot reasonably be on the ground of economy, for the difference in expense is slight, when you recollect that the gold when you are done with it is sold for nearly as much as it cost originally. I never, under any circumstances, use anything else. It is especially necessary with children, for whom most of this work is done, that the appliance be made as attractive as possible. I should consider copper very objectionable for this purpose. It would make the fingers smell badly, the mouth taste badly, and children would throw it away because it was disagreeable.

The subject was then passed.

Cairo was selected as the place for the next meeting.

The officers elected for the ensuing year were:

*President*—C. B. Rohland, Alton, Ill.

*Vice-President*—Chas. Henry, Jacksonville.

*Secretary*—Garrett Newkirk, Chicago.

*Treasurer*—T. W. Pritchett, Whitehall.

*Librarian*—W. B. Ames, Chicago.

*Executive Committee*—W. H. Taggart, P. J. Kester, J. Cormony.

(TO BE CONTINUED.)

